# Outcomes of high-dose levofloxacin therapy remain bound to the levofloxacin minimum inhibitory concentration in complicated urinary tract infections

**DOI:** 10.1186/s12879-016-2057-2

**Published:** 2016-11-25

**Authors:** Eliana S. Armstrong, Janelle A. Mikulca, Daniel J. Cloutier, Caleb A. Bliss, Judith N. Steenbergen

**Affiliations:** Merck & Co., Inc., 2000 Galloping Hill Road, Kenilworth, NJ 07033 USA

**Keywords:** Ceftolozane/tazobactam, cUTI, Fluoroquinolones, Levofloxacin, Resistance

## Abstract

**Background:**

Fluoroquinolones are a guideline-recommended therapy for complicated urinary tract infections, including pyelonephritis. Elevated drug concentrations of fluoroquinolones in the urine and therapy with high-dose levofloxacin are believed to overcome resistance and effectively treat infections caused by resistant bacteria. The ASPECT-cUTI phase 3 clinical trial (ClinicalTrials.gov, NCT01345929 and NCT01345955, both registered April 28, 2011) provided an opportunity to test this hypothesis by examining the clinical and microbiological outcomes of high-dose levofloxacin treatment by levofloxacin minimum inhibitory concentration.

**Methods:**

Patients were randomly assigned 1:1 to ceftolozane/tazobactam (1.5 g intravenous every 8 h) or levofloxacin (750 mg intravenous once daily) for 7 days of therapy. The ASPECT-cUTI study provided data on 370 patients with at least one isolate of Enterobacteriaceae at baseline who were treated with levofloxacin. Outcomes were assessed at the test-of-cure (5–9 days after treatment) and late follow-up (21–42 days after treatment) visits in the microbiologically evaluable population (*N* = 327).

**Results:**

Test-of-cure clinical cure rates above 90% were observed at minimum inhibitory concentrations ≤4 μg/mL. Microbiological eradication rates were consistently >90% at levofloxacin minimum inhibitory concentrations ≤0.06 μg/mL. Lack of eradication of causative pathogens at the test-of-cure visit increased the likelihood of relapse by the late follow-up visit.

**Conclusions:**

Results from this study do not support levofloxacin therapy for complicated urinary tract infections caused by organisms with levofloxacin minimum inhibitory concentrations ≥4 μg/mL.

**Trial registration:**

ClinicalTrials.gov, NCT01345929 and NCT01345955

**Electronic supplementary material:**

The online version of this article (doi:10.1186/s12879-016-2057-2) contains supplementary material, which is available to authorized users.

## Background

Complicated urinary tract infection (cUTI) is associated with significant morbidity and increased health care costs, particularly as resistance to first-line antimicrobial agents has become widespread [1, 2]. Hospitalization for cUTIs caused by Gram-negative bacteria (typically Enterobacteriaceae such as *Escherichia coli*) in the United States increased by approximately 50% from 2000 to 2009, whereas the incidence of infections caused by extended-spectrum β-lactamase (ESBL)–positive organisms increased by approximately 300% in the same time period [3].

Fluoroquinolones such as levofloxacin have historically been an attractive therapy for cUTI (including pyelonephritis) because of their high drug concentrations in the urine and their demonstrated clinical efficacy [4]. However, their future usefulness is threatened by alarmingly high rates of fluoroquinolone resistance, often occurring in combination with other resistance mechanisms including ESBL production. A surveillance study of 24 US hospitals found that less than 70% of non-ESBL–producing isolates of *E. coli* were susceptible to ciprofloxacin or levofloxacin, and this number fell to less than 10% for ESBL-producing strains [5]. The challenge of fluoroquinolone resistance is even greater in other regions of the world [6]. Resistance to fluoroquinolones can occur through a combination of point mutations in the target gyrase and topoisomerase genes, plasmid-mediated mechanisms (eg, qnr), and upregulated efflux of the drug [7, 8]. The spread of these resistance mechanisms has been facilitated by their strong association with the worldwide pandemic clone of *E. coli*, ST-131 [9].

In the clinical management of cUTI, it remains common practice to treat patients empirically, at least during the 2- to 3-day time period required to obtain standard culture results [10, 11]. As resistance increases, recommendations either to avoid empiric use of fluoroquinolones or to use these antibacterials judiciously based on local surveillance data are becoming more prevalent [12, 13]. Guidance directed toward uncomplicated urinary tract infections, published by the Infectious Diseases Society of America and the European Society for Clinical Microbiology and Infectious Diseases, supports a 3-day course of fluoroquinolone therapy, but only in regions where the resistance rate is lower than 10% [14].

Previous clinical studies have concluded that high urinary tract concentrations of fluoroquinolones are sufficient to allow successful outcomes for nonsusceptible isolates. One large *post hoc* analysis examined a population in whom the level of fluoroquinolone resistance was relatively low (<10%) [15]. Patients received levofloxacin (750 mg intravenously) or ciprofloxacin (either 400 mg intravenously or 500 mg orally) for 10 days. Forty-two fluoroquinolone-resistant isolates (levofloxacin minimum inhibitory concentration [MIC] ≥8 μg/mL and ciprofloxacin MIC ≥4 μg/mL) were reported. Six isolates had levofloxacin or ciprofloxacin MICs ranging from 8 to 32 μg/mL and were eradicated with treatment, and 13 isolates with fluoroquinolone MICs >32 μg/mL persisted. The outcomes of the remaining 23 isolates were not presented. A smaller study of a 500-mg dose of levofloxacin conducted in a setting of higher levofloxacin resistance calculated a 90% probability of microbiological eradication of Gram-negative bacilli at an MIC of 2 μg/mL. Eradication was evaluated only 2 days after therapy, however, a time when levofloxacin may still be present in the urine and suppressing the growth of organisms that could result in a different microbiological outcome after the drug has cleared [16]. In vitro urinary bactericidal titer experiments demonstrated that the concentration of levofloxacin in urine following a 750-mg dose was bactericidal to *E. coli* isolates with MICs ≤32 μg/mL [17].

In this exploratory analysis, we examined the clinical and microbiological outcomes of treatment with high-dose, extended-duration levofloxacin (750 mg for 7 days) by levofloxacin MIC in a large sample of patients with cUTI caused by Enterobacteriaceae in the ASPECT-cUTI phase 3 clinical trial.

## Methods

### Study population

ASPECT-cUTI (ClinicalTrials.gov, NCT01345929 and NCT01345955) was a large, global, phase 3 program that evaluated the efficacy and safety of ceftolozane/tazobactam versus levofloxacin for the treatment of adult hospital patients with cUTI, including pyelonephritis [18]. The trials were approved by appropriate regulatory agencies and local institutional review boards (IRB) and were conducted in accordance with International Conference on Harmonisation/Good Clinical Practice guidelines and the principles of the Declaration of Helsinki. All patients provided written informed consent. Patients with cUTI (defined in [18]) were randomly assigned 1:1 to ceftolozane/tazobactam (1.5 g intravenous every 8 h) or levofloxacin (750 mg intravenous once daily) for 7 days of therapy. Patients were included in the microbiological modified intent-to-treat (mMITT) population if they received any amount of study drug and had at least one but not more than two causative uropathogen(s) growing at ≥10^5^ CFU/mL from a study-qualifying pretreatment baseline urine specimen. The microbiologically evaluable (ME) population was the subset of the mMITT population who adhered to study procedures and had interpretable urine culture results at the test-of-cure (TOC) visit (5–9 days after the last dose of study drug).

The ASPECT-cUTI study provided data on 370 patients in the mMITT population and 327 patients in the ME population who had ≤1 isolate of Enterobacteriaceae at baseline and were treated with levofloxacin. A total of 333 Enterobacteriaceae were isolated from 327 patients in the ME population. Susceptibility data were available for 313 of these isolates.

### Levofloxacin efficacy analysis

An exploratory analysis of levofloxacin-treated patients with an Enterobacteriaceae isolate and characterized levofloxacin susceptibility from the screening visit in ASPECT-cUTI was conducted to evaluate the microbiological and clinical outcomes at the TOC and late follow-up (LFU) visits (21–42 days after the last dose of study drug). The ME population was selected for analysis to exclude patients with indeterminate outcomes. Clinical cure at the TOC visit was defined as complete resolution, substantial improvement (ie, reduction in severity of all baseline signs and symptoms), or return to premorbid signs and symptoms without the need for additional antibiotic therapy. Microbiological eradication was defined as a TOC urine culture with <10^4^ CFU/mL of the baseline uropathogen. The per-pathogen microbiological and clinical outcomes at the TOC visit were stratified by levofloxacin MIC of each baseline infecting organism. Clinical outcomes for patients who were classified as clinical cures at the TOC visit and who returned for the LFU visit were classified as sustained, indeterminate, or relapsed based on the sustained resolution or relapse in clinical signs and symptoms of cUTI that were absent at the TOC visit. Relative proportions of each of these categories were examined for patients whose infections were microbiologically eradicated versus those who had persisting positive cultures at the TOC visit.

### Susceptibility testing

Susceptibility and quality control testing of study isolates was performed by ICON Laboratories (Farmingdale, NY) in accordance with Clinical and Laboratory Standards Institute (CLSI) guidelines. Quality control testing was performed concurrent with testing of clinical study isolates. Interpretation of susceptibility test results and acceptable quality control ranges were based on CLSI document M100-S22 [19]. Study isolates initially characterized as levofloxacin-resistant (MIC >4 μg/mL) [19] were retested to determine the levofloxacin MIC end point up to 256 μg/mL.

### Statistical analysis

Descriptive statistics were used to summarize the demographics, baseline characteristics, clinical outcomes, microbiological outcomes, and genotypes of patients, and 95% confidence intervals (CIs) were calculated by the Wilson score methodology to compare the relapse rates at LFU. Statistical analyses were performed using SAS version 9.1 (SAS Institute Inc., Cary, NC).

## Results

Demographics and baseline clinical characteristics of 370 levofloxacin-treated patients with ≥1 isolate each of Enterobacteriaceae identified at baseline in the mMITT population are shown in Table 1. Most patients had pyelonephritis (82.7%) and most were enrolled in Europe (74.9%); 24.3% were 65 years of age or older.

**Table 1:** Characteristics of levofloxacin-treated patients with ≥1 isolate of Enterobacteriaceae identified at baseline (mMITT population)

Characteristic	Levofloxacin *N* = 370
Age, years	
Mean (SD)	48.0 (20.26)
Range, *n* (%)	18–87
≥ 65–< 75	47 (12.7)
≥ 75	43 (11.6)
Sex, *n* (%)	
Male	82 (22.2)
Female	288 (77.8)
Race, *n* (%)	
White	317 (85.7)
Black or African American	6 (1.6)
Asian	30 (8.1)
Other	17 (4.6)
Region, *n* (%)	
Eastern Europe	277 (74.9)
Western Europe	0
North America	9 (2.4)
South America	39 (10.5)
Rest of world	45 (12.2)
Diagnosis, *n* (%)	
Pyelonephritis	306 (82.7)
cLUTI	64 (17.3)
Creatinine clearance, mL/min, *n* (%)	
Normal (≥80)	250 (67.6)
Mild impairment (≥50–< 80)	92 (24.9)
Moderate impairment (≥30–< 50)	27 (7.3)
Severe impairment (<30)	1 (0.3)

*cLUTI* complicated lower urinary tract infection, *mMITT* microbiological modified intent-to-treat, *SD* standard deviation

Clinical and microbiological outcomes of levofloxacin treatment stratified by levofloxacin MICs are listed in Table 2. High rates of clinical cure (90–100%) were observed at levofloxacin MICs ≤4 μg/mL. Rates of microbiological eradication were also consistently high (>90%) at levofloxacin MICs ≤0.06 μg/mL. However, at levofloxacin MICs >0.06 μg/mL, a trend toward decreasing eradication rates was observed.

**Table 2:** Microbiological eradication and clinical cure following levofloxacin treatment stratified by MIC (Enterobacteriaceae isolates, ME population)

Baseline levofloxacin MIC, μg/mL	Microbiological eradication, *n*/*N* (%)	Clinical cure, *n*/*N* (%)
≤0.015	9/10 (90.0)	10/10 (100)
0.03	120/126 (95.2)	124/126 (98.4)
0.06	38/40 (95.0)	37/40 (92.5)
0.125	9/11 (81.8)	10/11 (90.9)
0.25	6/8 (75.0)	8/8 (100)
0.5	14/17 (82.4)	17/17 (100)
1	5/6 (83.3)	6/6 (100)
2	1/1 (100)	0/1 (0)^a^
4	3/9 (33.3)	9/9 (100)
8	11/17 (64.7)	15/17 (88.2)
16	20/44 (45.5)	36/44 (81.8)
32	4/13 (30.8)	11/13 (84.6)
64	1/6 (16.7)	4/6 (66.7)
128	0/1 (0)	0/1 (0)

*ME* microbiologically evaluable, *MIC* minimum inhibitory concentration, *n* number of isolates assigned to an outcome of eradication or clinical cure, *N* number of isolates at each levofloxacin MIC

Four *E. coli* isolates were not retested to determine MIC end points and were excluded from this analysis. Five patients had 2 Enterobacteriaceae isolates identified at baseline. Four patients had a clinical response of cure, and 1 patient had a clinical response of failure. Clinical response for these patients was counted once for each isolate at its respective MIC

^*a*^ This isolate was present in combination with a second isolate that had a levofloxacin MIC of 16 μg/mL; the patient’s clinical response was failure

A higher percentage of patients with persistent infection at the TOC visit experienced relapse by the LFU visit than did patients whose infections were eradicated at the TOC visit (18.5% vs 1.3%; difference, 17.2%; 95% CI, 8.7–29.6%) (Table 3).

**Table 3:** Impact of microbiological outcome at TOC on clinical outcome at LFU (ME population)

Microbiological outcome at TOC visit	Clinical outcome at LFU visit			% Difference in relapse between persisted and eradicated (95% CI)
	Sustained	Indeterminate	Relapse	
Eradicated, *n*/*N* (%)	225/229 (98.3)	1/229 (0.4)	3/229 (1.3)	17.2 (8.7–29.6)
Persisted, *n*/*N* (%)	42/54 (77.8)	2/54 (3.7)	10/54 (18.5)	

*CI* confidence interval, *LFU* late follow-up, *ME* microbiologically evaluable, *TOC* test-of-cure

Four patients had 2 Enterobacteriaceae isolates identified at baseline and a clinical response of cure at the TOC visit. These patients were counted once for this analysis by assignment to the “persisted at TOC group” if 1 isolate persisted (1 patient) and to the “eradicated at TOC group” if both isolates were eradicated (3 patients). All 4 experienced sustained response at the LFU visit

## Discussion and conclusions

In several recent and ongoing clinical trials in cUTI, levofloxacin has been used as the comparator against such agents as doripenem [20], plazomicin (NCT01096849), ceftolozane/tazobactam [18], and eravacycline (NCT01978938). A recent meta-analysis examined multiple cUTI trials before ASPECT-cUTI and identified microbiological eradication rates at the TOC visit in the microbiological intent-to-treat population of 81% for doripenem, 79% for levofloxacin, and 80.5% for imipenem-cilastatin, confirming that levofloxacin has a place in the armamentarium for cUTI [21]. In an era of increasing and evolving antibiotic resistance, however, decisions about clinical usefulness must be made at the local hospital and individual patient level [22].

High-dose levofloxacin treatment (750 mg once daily for 7 days) in ASPECT-cUTI resulted in clinical cure for the more than 90% of patients with Enterobacteriaceae infections who had MICs ≤4 μg/mL. Nevertheless, persistence of the baseline pathogen became more frequent as the MIC increased, with an eradication rate of <90% observed at an MIC of only 0.125 μg/mL. A similar decrease in the microbiological eradication rate with levofloxacin treatment above a levofloxacin MIC of 0.06 μg/mL was observed during the phase 3 doripenem studies, though the total numbers of patients treated was smaller [20].

The clinical significance of failure to eradicate the causative pathogen while resolving clinical symptoms is unknown, but multiple studies have shown that fluoroquinolone resistance is strongly correlated with persistence of symptoms [23] and recurrence of infection [24]. The results presented in this study show that, as expected, an increase in levofloxacin MIC was correlated with a decrease in microbiological eradication rate with levofloxacin treatment. Clinical cure without microbiological eradication of the causative pathogen was more likely (difference, 17.2%; 95% CI, 8.7–29.6%) to result in a relapse of cUTI at the LFU visit. The levofloxacin-resistant isolates from this study had high levofloxacin MICs that were associated with common and highly conserved fluoroquinolone resistance mechanisms, most typically point mutations in gyrA and parC (data not shown).

Pharmacokinetic/pharmacodynamic (PK/PD) targets for fluoroquinolone treatment in cUTI have been studied in depth. Typically, AUC/MIC ratios of 100 or C_max_/MIC ratios of 10 are associated with good clinical outcomes [25], though 90% microbiological eradication of Gram-negative cUTI pathogens has been reported at targets as low as 31.46 and 2.74, respectively [16]. Data on the pharmacokinetics of levofloxacin in either serum or urine were not obtained during ASPECT-cUTI; therefore, direct analysis of these outcomes as they relate to the levofloxacin concentration is not possible. Administration of single 750-mg doses of levofloxacin to 10 healthy adults was reported to produce AUC and C_max_ values of 93 ± 15 μg · h/mL and 7.6 ± 1.2 μg/mL in serum and 7328 ± 3237 μg · h/mL and 620 ± 324 μg/mL in urine, respectively [17]. Conservatively applying the lower bound of the PK ranges and the higher PK/PD targets predicts that levofloxacin MICs of 0.5 μg/mL should be treatable in serum. In urine, the value is between 16 and 32 μg/mL, depending on whether the C_max_ or the AUC parameter is used. However, elevated MIC treatment targets based on urinary concentrations are not supported by the outcomes in this study, which is the first to examine a population with high levels of levofloxacin resistance using a stringent criterion for microbiological eradication.

In summary, these clinical data demonstrate that high-dose, extended-duration levofloxacin treatment (750 mg for 7 days) of patients with cUTIs was less likely to be successful when the MIC of the infecting organism was ≥4 μg/mL and less likely to be sustainable when the MIC was >0.06 μg/mL.

## Additional file

Additional file 1: List of Institutional Review Boards (IRB) and Independent Ethics Committees (IEC). (DOCX 13 kb)

## Abbreviations

CFU: Colony-forming unit
CI: Confidence interval
CLSI: Clinical and Laboratory Standards Institute
cUTI: Complicated urinary tract infection
ESBL: Extended-spectrum β-lactamase
LFU: Late follow-up
ME: Microbiologically evaluable
MIC: Minimum inhibitory concentration
mMITT: Microbiological modified intent-to-treat
PD: Pharmacodynamic
PK: Pharmacokinetic
TOC: Test-of-cure

## References

[1] Klevens RM, Edwards JR, Gaynes RP (2008). The impact of antimicrobial-resistant, health care-associated infections on mortality in the United States. Clin Infect Dis.

[2] Sader HS, Flamm RK, Jones RN (2014). Frequency of occurrence and antimicrobial susceptibility of Gram-negative bacteremia isolates in patients with urinary tract infection: results from United States and European hospitals (2009–2011). J Chemother.

[3] Zilberberg MD, Shorr AF (2013). Secular trends in Gram-negative resistance among urinary tract infection hospitalizations in the United States, 2000–2009. Infect Control Hosp Epidemiol.

[4] Rafat C, Debrix I, Hertig A (2013). Levofloxacin for the treatment of pyelonephritis. Expert Opin Pharmacother.

[5] Bouchillon SK, Badal RE, Hoban DJ, Hawser SP (2013). Antimicrobial susceptibility of inpatient urinary tract isolates of Gram-negative bacilli in the United States: results from the study for monitoring antimicrobial resistance trends (SMART) program: 2009–2011. Clin Ther.

[6] Hsueh PR, Hoban DJ, Carmeli Y, Chen SY, Desikan S, Alejandria M, Ko WC, Binh TQ (2011). Consensus review of the epidemiology and appropriate antimicrobial therapy of complicated urinary tract infections in Asia-Pacific region. J Infect.

[7] Hooper DC (2000). Mechanisms of action and resistance of older and newer fluoroquinolones. Clin Infect Dis.

[8] Tran JH, Jacoby GA (2002). Mechanism of plasmid-mediated quinolone resistance. Proc Natl Acad Sci U S A.

[9] Rogers BA, Sidjabat HE, Paterson DL (2011). *Escherichia coli* O25b-ST131: a pandemic, multiresistant, community-associated strain. J Antimicrob Chemother.

[10] Nicolle LE (2005). Complicated urinary tract infection in adults. Can J Infect Dis Med Microbiol.

[11] Pallett A, Hand K. Complicated urinary tract infections: practical solutions for the treatment of multiresistant Gram-negative bacteria. J Antimicrob Chemother. 2010;65 suppl 3:iii25–33. 10.1093/jac/dkq298 20876625

[12] Bader MS, Hawboldt J, Brooks A (2010). Management of complicated urinary tract infections in the era of antimicrobial resistance. Postgrad Med.

[13] Koningstein M, Van der Bij AK, De Kraker ME, Monen JC, Muilwijk J, De Greeff SC, Geerlings SE, Leverstein-van Hall MA, ISIS-AR Study Group (2014). Recommendations for the empirical treatment of complicated urinary tract infections using surveillance data on antimicrobial resistance in the Netherlands. PLoS One.

[14] Gupta K, Hooton TM, Naber KG, Wullt B, Colgan R, Miller LG, Moran GJ, Nicolle LE, Raz R, Schaeffer AJ, Soper DE (2011). Infectious Diseases Society of America; European Society for Microbiology and Infectious Diseases. International clinical practice guidelines for the treatment of acute uncomplicated cystitis and pyelonephritis in women: a 2010 update by the Infectious Diseases Society of America and the European Society for Microbiology and Infectious Diseases. Clin Infect Dis.

[15] Peterson J, Kaul S, Khashab M, Fisher A, Kahn JB (2007). Identification and pretherapy susceptibility of pathogens in patients with complicated urinary tract infection or acute pyelonephritis enrolled in a clinical study in the United States from November 2004 through April 2006. Clin Ther.

[16] Deguchi T, Nakane K, Yasuda M, Shimizu T, Monden K, Arakawa S, Matsumoto T (2010). Microbiological outcome of complicated urinary tract infections treated with levofloxacin: a pharmacokinetic/pharmacodynamic analysis. Int J Antimicrob Agents.

[17] Stein GE, Schooley SL, Nicolau DP (2008). Urinary bactericidal activity of single doses (250, 500, 750 and 1000 mg) of levofloxacin against fluoroquinolone-resistant strains of *Escherichia coli*. Int J Antimicrob Agents.

[18] Wagenlehner FM, Umeh O, Steenbergen J, Yuan G, Darouiche RO (2015). Ceftolozane-tazobactam compared with levofloxacin in the treatment of complicated urinary-tract infections, including pyelonephritis: a randomised, double-blind, phase 3 trial (ASPECT-cUTI). Lancet.

[19] Clinical and Laboratory Standards Institute. Performance Standards for Antimicrobial Susceptibility Testing: Twenty-Second Informational Supplement M100-S22. Wayne: Clinical and Laboratory Standards Institute; 2012.

[20] Naber KG, Llorens L, Kaniga K, Kotey P, Hedrich D, Redman R (2009). Intravenous doripenem at 500 milligrams versus levofloxacin at 250 milligrams, with an option to switch to oral therapy, for treatment of complicated lower urinary tract infection and pyelonephritis. Antimicrob Agents Chemother.

[21] Singh KP, Li G, Mitrani-Gold FS, Kurtinecz M, Wetherington J, Tomayko JF, Mundy LM (2013). Systematic review and meta-analysis of antimicrobial treatment effect estimation in complicated urinary tract infection. Antimicrob Agents Chemother.

[22] Chen YH, Ko WC, Hsueh PR (2012). The role of fluoroquinolones in the management of urinary tract infections in areas with high rates of fluoroquinolone-resistant uropathogens. Eur J Clin Microbiol Infect Dis.

[23] Can F, Azap OK, Seref C, Ispir P, Arslan H, Ergonul O (2015). Emerging *Escherichia coli* O25b/ST131 clone predicts treatment failure in urinary tract infections. Clin Infect Dis.

[24] Bodro M, Sanclemente G, Lipperheide I, Allali M, Marco F, Bosch J, Cofan F, Ricart MJ, Esforzado N, Oppenheimer F, Moreno A, Cervera C (2015). Impact of antibiotic resistance on the development of recurrent and relapsing symptomatic urinary tract infection in kidney recipients. Am J Transplant.

[25] Schentag JJ (2000). Clinical pharmacology of the fluoroquinolones: studies in human dynamic/kinetic models. Clin Infect Dis.

