# Supplementary material for: Outcomes of high-dose levofloxacin therapy remain bound to the levofloxacin minimum inhibitory concentration in complicated urinary tract infections
Source: BMC Infect Dis. 2016 Nov 25;16:710. doi: 10.1186/s12879-016-2057-2 (PMC5124239; doi:10.1186/s12879-016-2057-2)
Supplement: Additional file 1: — List of Institutional Review Boards (IRB) and Independent Ethics Committees (IEC). (DOCX 13 kb) [file 12879_2016_2057_MOESM1_ESM.docx]

**Supplementary Data**

***List of Institutional Review Boards (IRB) and Independent Ethics Committees (IEC)***

- DeKalb Medical IRB, 2701 North Decatur Road, Decatur, GA 30033, USA
- Western Institutional Review Board, 3535 Seventh Ave, SW Olympia, WA 98502-5010, USA
- St. Vincent Hospital Institutional Review Board, 8402 Harcourt Road, Indianapolis, IN 46260, USA
- Ethikkommission des FB Medizin, Gaffkystr. 11c, D-35385 Giessen, Germany
- Komisja Bioetyczna przy Centrum Medycznym, Ksztalcenia Podyplomowego w Warszawie, Ul. Marymoncka 99/103, Warszawa 01-813, Poland
